# The mediating effect of handgrip strength on the association between sleep duration and basal metabolic rate in middle-aged and older adults: evidence from the China health and retirement longitudinal study

**DOI:** 10.3389/fpubh.2025.1567396

**Published:** 2025-07-17

**Authors:** Xinxiang Sun, Changqing Li, Hong Ding, Hao Zhou, Ning Bai, Xiaojiang Zhao

**Affiliations:** ^1^School of Sports Leisure, Shandong Sport University, Jinan, China; ^2^Department of Physical Education and Arts, Bengbu Medical University, Bengbu, China; ^3^Department of Physical Education, Anhui Finance and Economics University, Bengbu, China

**Keywords:** sleep duration, basal metabolic rate, handgrip strength, middle-aged and older adults, CHARLS

## Abstract

**Background:**

Anomalies in basal metabolic rate (BMR) among middle-aged and older populations can lead to various metabolism-related diseases, presenting a significant global public health challenge. The association and mechanism between sleep duration and BMR remain unclear. This study aimed to investigate the mediating role of handgrip strength in the relationship between sleep duration and BMR.

**Methods:**

The study utilized data from the 2015 China Health and Retirement Longitudinal Study (CHARLS), comprising 10,161 participants aged 45 and older. The mediating effect of handgrip strength on the relationship between sleep duration and BMR was analyzed using linear regression model and bootstrap method.

**Results:**

After controlling for confounding variables, a positive correlation was observed between sleep duration and BMR, with standardized regression coefficients (*β*) of 1.65, 95% confidence interval (CI) ranging from 0.63 to 2.70, and a significance level of *p* = 0.002. Grip strength was positively correlated with BMR, *β* was 4.63, 95% CI: 4.34 to 5.91, *p* < 0.001. Handgrip strength mediates 22.42% of the total effect linking sleep duration to BMR. The mediating effect was 0.37, 95% CI: 0.07–0.67.

**Conclusion:**

The study identified significant positive correlations between sleep duration and BMR, as well as handgrip strength and BMR, with handgrip strength mediating the relationship between sleep duration and BMR.

## 1 Introduction

The global discussion often focuses on population aging. The World Health Organization (WHO) anticipates a seven- to eight-fold rise in the older population in developing nations. By 2025, China’s population aged 60 and above is expected to exceed 300 million (1). Basal metabolic rate (BMR) is the minimum energy required to maintain essential physiological functions at rest (2). Factors such as age, sex, body weight, body composition, and ambient temperature affect BMR (3), which tends to be lower in middle-aged and older individuals (4). Abnormal BMR can negatively impact health, increasing vulnerability to infectious diseases, metabolic disorders, cancer, and affecting weight management success (3, 5–7). Individuals with atypical BMR may experience reduced productivity, negatively affecting labor efficiency and economic output, which increases the economic burden (8, 9). Additionally, they may encounter stigmatization and mental health issues, leading to discrimination or social isolation (10, 11). Thus, implementing preventive measures and early interventions is crucial to mitigate the effects of abnormal BMR in middle-aged and older populations.

Sleep duration is crucial for the health of middle-aged and older individuals (12), influenced by genetic, environmental, and physiological factors, such as inflammation (13, 14), which may also affect BMR abnormalities. Research indicates a significant link between sleep duration and BMR. sleep restriction lowers resting metabolic rate, a component of BMR (15). A meta-analysis suggests that both short and long sleep durations may increase the risk of metabolic syndrome, implying a link between sleep length and metabolic health, with sleep potentially affecting BMR through metabolic syndrome components (16). However, another study found that short sleep duration does not significantly impact total daily energy expenditure, including BMR (17). Thus, the precise relationship between sleep duration and BMR, along with the underlying mechanisms, requires further investigation.

Handgrip strength is a recognized indicator of physical performance and a predictor of health outcomes (18). It is strongly associated with sleep duration and mortality risk (19, 20). Previous research indicates that poor sleep quality and short sleep duration correlate with reduced muscle strength, including handgrip strength, in older adults (21), emphasizing the role of sleep in preserving muscle mass and function. Additionally, another study explored the relationship between sleep duration and handgrip strength, indicating that insufficient sleep could lead to a decline in muscle strength over time (22). Research indicates that reduced grip strength is linked to higher comorbidity risk, especially in older women, highlighting the importance of maintaining muscle strength for metabolic health and a healthy BMR (23). This relationship persists across different populations and remains significant after adjusting for confounders (24, 25). Insufficient sleep may lead to a decrease in handgrip strength, thereby reducing BMR. However, the role of handgrip strength in the mediating relationship between sleep duration and BMR remains unclear. Consequently, we undertook a nationally representative cross-sectional analysis utilizing data from the China Health and Retirement Longitudinal Study (CHARLS) database. The objective of this research was to investigate the relationships between sleep duration, handgrip strength, and BMR within Chinese middle-aged and older adults examines whether handgrip strength mediates this relationship.

## 2 Materials and methods

### 2.1 Study design and participants

This study utilized a cross-sectional design, drawing upon data from the CHARLS. Which is a national longitudinal survey aimed at Chinese residents aged 45 and above, meticulously designed to produce a high-quality, representative, and publicly accessible micro-database. This database offers extensive information on middle-aged and older individuals. Participants were chosen from 28 Chinese provinces using a multi-stage probability proportional to size sampling technique. Comprehensive details regarding CHARLS have been previously documented (26). The CHARLS Dataset can be downloaded from its official website. Participants provided written informed consent and the study was ethically approved by Peking University’s Biomedical Ethics Review Board (IRB00001052-11015).

**Figure 1:**
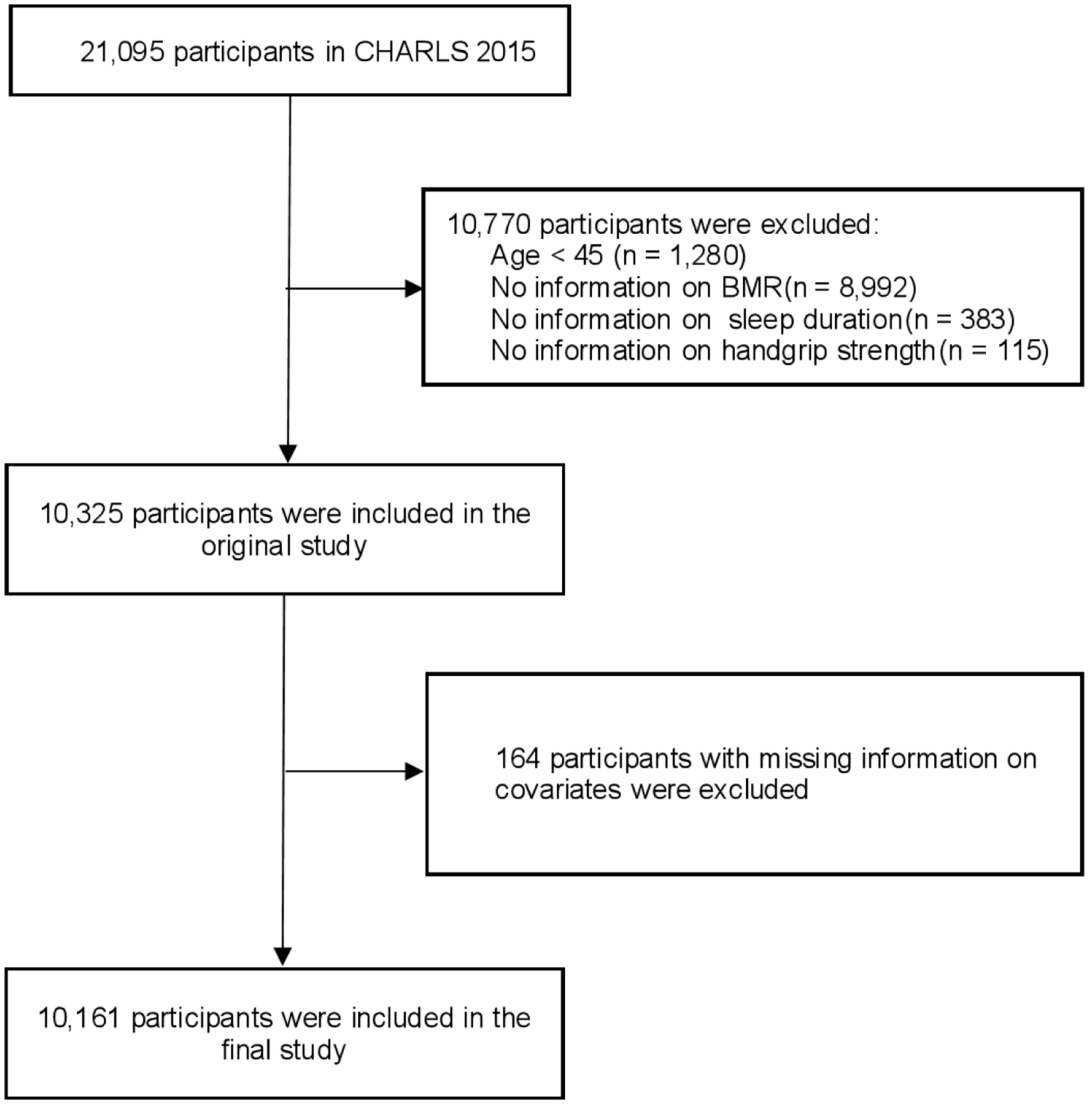
Flowchart of the participants selection process. CHARLS, China health and retirement longitudinal study, BMR, Basal metabolic rate.

This study analyzed baseline data from 21,095 participants collected in 2015. Initially, 1,280 participants under the age of 45 were excluded. Subsequently, 8,992 individuals without BMR data, 383 without sleep duration data, and 115 without grip strength data were also excluded. Additionally, 164 participants were excluded due to missing covariate data. Supplementary Table 1 displays the traits of the participants who were excluded. Consequently, the final sample comprised 10,161 participants. Figure 1 presents a flowchart detailing the exclusion criteria.

### 2.2 Assessments

#### 2.2.1 Sleep duration

Sleep duration was evaluated with the question: “Over the past month, how many hours of actual sleep did you obtain at night on average?” This aimed to determine the average sleep hours, accounting for both workdays and days off. This methodology enables a systematic analysis of sleep patterns in relation to health outcomes (27).

#### 2.2.2 Handgrip strength

Handgrip strength was evaluated at baseline using a hydraulic handgrip dynamometer. Each participant’s hands were measured twice on both the left and right sides. For statistical analysis, the highest value among the four measurements was utilized as a continuous variable (28, 29).

#### 2.2.3 Basal metabolic rate

BMR was calculated using the Mifflin St Jeor method (30). The Mifflin-St Jeor equations estimate BMR as follows: for males, BMR = 10 × weight (kg) + 6.25 × height (cm) − 5 × age (years) + 5; for females, BMR = 10 × weight (kg) + 6.25 × height (cm) − 5 × age (years) − 161.

#### 2.2.4 Control variables

Building upon existing literature and clinical evidence (31, 32), this study incorporated sociodemographic characteristics, lifestyle habits, personal health status as covariates. Sociodemographic characteristics variables encompass age, gender, Education levels, marital status, and usual residence. Gender was defined as male or female. Educational attainment was categorized into three levels: “illiteracy,” “primary or middle school,” and “high school and above. “Marital status was classified as “married” for individuals cohabitating with a spouse, and “unmarried” for those who were separated, divorced, widowed, or single. Residence was dichotomized into rural or urban categories. Lifestyle habits encompassed smoking and drinking status, each classified as “yes” or “no.” The personal health status encompassed body mass index (BMI) and drinking status14 self-reported non-communicable conditions, 14 self-reported non-communicable conditions included hypertension, dyslipidemia, diabetes, cancer, chronic pulmonary disease, liver disease, myocardial infarction, cerebrovascular accident, renal disease, asthma, psychiatric disorders, gastrointestinal disease, cognitive disorders, and arthritis. Additionally, BMI was classified into categories: underweight, normal weight, Overweight, and Obesity.

### 2.3 Statistical analysis

This study conducted all analyses using R version 4.3.3. Sample characteristics were summarized using means and standard deviations for continuous variables, and frequencies and percentages for categorical variables. A Shapiro–Wilk test was employed to assess the normality of the continuous variables’ distribution Initially, Spearman’s correlation was employed to assess the relationships among the primary variables. A multiple linear regression examined the associations between sleep duration, handgrip strength, and BMR, with the standardized regression coefficients (*β*) and 95% confidence interval (95% CI) serving to quantify the strength of these associations (33). Four adjusting progressive models were applied: an unadjusted model, Model 1 (adjusted for age, sex, educational level, and marital status), Model 2 (Model 1 plus adjustments for smoking status and drinking status), and Model 3 (adjusted for all covariates). Subsequently, using the adjustment variables from Model 3, restricted cubic spline (RCS) fitting was utilized to investigate potential nonlinear associations between sleep duration and BMR, as well as between the handgrip strength and BMR. nflection points (IP) and likelihood ratio tests (LRT) were employed to evaluate the threshold effects of sleep duration on BMR. Finally, we intend to further investigate the role of handgrip strength in this association. To this end, we developed a mediation effect model to assess the mediating role of handgrip strength in the pathway of association between sleep duration and BMR. The mediating effect’s significance was assessed through a bootstrap resampling method with 1,000 resamples (34). Statistical significance determined at a two-tailed *p*-value of less than 0.05.

## 3 Result

### 3.1 Baseline characteristics of the study participants

Participants were divided into male and female groups, with their characteristics detailed in Table 1. The study included 10,161 participants (mean age = 66.6 years, SD = 5.3), consisting of 4,888 men (mean age = 60.4 years, SD = 9.6) and 5,273 women (mean age = 59.5 years, SD = 9.4). The male cohort exhibited significantly higher marriage rates and educational attainment (*p* < 0.001). A significant percentage (61.2%) of middle-aged and older participants lived in urban regions. Significant differences in alcohol consumption, smoking habits, chronic medical conditions prevalence, and BMI were found between male and female groups (*p* < 0.001). Men averaged 6.6 h of sleep per day (SD = 1.8), while women averaged 6.3 h (SD = 2.0), with a statistically significant difference (*p* < 0.001). Mean grip strength was recorded at 38.1 kg (SD = 8.7) for men and 25.4 kg (SD = 6.6) for women (*p* < 0.001). The study found a significant difference in mean BMR between men (1,374.8 kJ/day, SD = 167) and women (1,075.2 kJ/day, SD = 153.6) with *p* < 0.001.

**Table 1:** Characteristics of the study participants according to sex.

Variables	Total (*n* = 10,161)	Male (*n* = 4,888)	Female (*n* = 5,273)	*p*
Age, Mean ± SD	59.9 ± 9.5	60.4 ± 9.6	59.5 ± 9.4	< 0.001
Marital status, *n* (%)				< 0.001
Married	8,954 (88.1)	4,456 (91.2)	4,498 (85.3)	
Unmarried	1,207 (11.9)	432 (8.8)	775 (14.7)	
Drinking, *n* (%)				< 0.001
No	5,626 (55.4)	891 (18.2)	4,735 (89.8)	
Yes	4,535 (44.6)	3,997 (81.8)	538 (10.2)	
Smoking, *n* (%)				< 0.001
No	5,445 (53.6)	1,312 (26.8)	4,133 (78.4)	
Yes	4,716 (46.4)	3,576 (73.2)	1,140 (21.6)	
Education levels, *n* (%)				< 0.001
Illiteracy	6,853 (67.4)	2,827 (57.8)	4,026 (76.4)	
Primary or middle school	2,156 (21.2)	1,307 (26.7)	849 (16.1)	
High school and above	1,152 (11.3)	754 (15.4)	398 (7.5)	
Residence, *n* (%)				0.049
Urban	6,217 (61.2)	3,039 (62.2)	3,178 (60.3)	
Rural	3,944 (38.8)	1849 (37.8)	2095 (39.7)	
BMI group, *n* (%)				< 0.001
Underweight	522 (5.1)	263 (5.4)	259 (4.9)	
Normal	5,804 (57.1)	2,992 (61.2)	2,812 (53.3)	
Overweight	3,223 (31.7)	1,425 (29.2)	1798 (34.1)	
Obesity	612 (6.0)	208 (4.3)	404 (7.7)	
Number of chronic conditions, *n* (%)				< 0.001
0	3,064 (30.2)	1,560 (31.9)	1,504 (28.5)	
1	2,414 (23.8)	1,219 (24.9)	1,195 (22.7)	
≥2	4,683 (46.1)	2,109 (43.1)	2,574 (48.8)	
Sleep duration (h/day), Mean ± SD	6.4 ± 1.9	6.6 ± 1.8	6.3 ± 2.0	< 0.001
Handgrip strength (kg), Mean ± SD	31.5 ± 10.0	38.1 ± 8.7	25.4 ± 6.6	< 0.001
BMR (kJ/day), Mean ± SD	1219.3 ± 219.2	1374.8 ± 167.0	1075.2 ± 153.6	< 0.001

BMI, body mass index; BMR, Basal metabolic rate.

### 3.2 The relationship between important variables

Table 2 presents the relationships between sleep duration, handgrip strength, and BMR. The research assessed the link between sleep duration and BMR among 10,161 participants. In the unadjusted model, the association between sleep duration and BMR showed a significant positive relationship, with a *β* of 12.04 (95% CI: 9.85–14.26, *p* < 0.001). In model 1, *β* was 1.46, 95% CI: (0.43–2.49); *p* = 0.006. In model 2, *β* = 1.51, 95% CI: (0.48–2.54); *p* = 0.004. In model 3, *β* was 1.65, 95% CI: (0.63–2.70); *p* = 0.002. This suggested that controlling for related factors did not significantly change the positive association between sleep duration and BMR. Handgrip strength was significantly associated with BMR in the unadjusted model (*β* = 16.07, 95% CI: 15.78.16.36, *p* < 0.001). In model 1, *β* was 4.56, 95% CI: (4.28–4.85); *p* < 0.001. In model 2, *β* was 4.55, 95% CI: (4.27–4.84); *p* < 0.001. In model 3, *β* was 4.63, 95% CI: (4.34–4.91); *p* < 0.001. The positive correlation between handgrip strength and BMR persisted significantly after controlling for relevant variables. In summary, both sleep duration and grip strength were significantly and positively associated with BMR. After accounting for various confounders, the effects of sleep duration and grip strength on BMR remained statistically significant and robust, despite variations in magnitude.

**Table 2:** Associations of sleep duration and handgrip strength with basal metabolic rate.

Variables	No	Unadjusted		Model 1		Model 2		Model 3	
		*β* (95% CI)	*p* value	*β* (95% CI)	*p* value	*β* (95% CI)	*p* value	*β* (95% CI)	*p* value
Sleep duration	10,161	12.04 (9.85 ~ 14.26)	<0.001	1.46 (0.43 ~ 2.49)	0.006	1.51 (0.48 ~ 2.54)	0.004	1.65 (0.63 ~ 2.70)	0.002
Handgrip strength	10,161	16.07 (15.78 ~ 16.36)	<0.001	4.56 (4.28 ~ 4.85)	<0.001	4.55 (4.27 ~ 4.84)	<0.001	4.63 (4.34 ~ 4.91)	<0.001

Model 1: adjusted for age, gender, educational level, marital status, and residence. Model 2: adjusted for model 1 + smoking status, drinking status, and BMI. Model 3: adjusted for model 2 + 14 chronic diseases. *β*, Standardized regression coefficients; 95% CI, 95% confidence interval.

**Figure 2:**
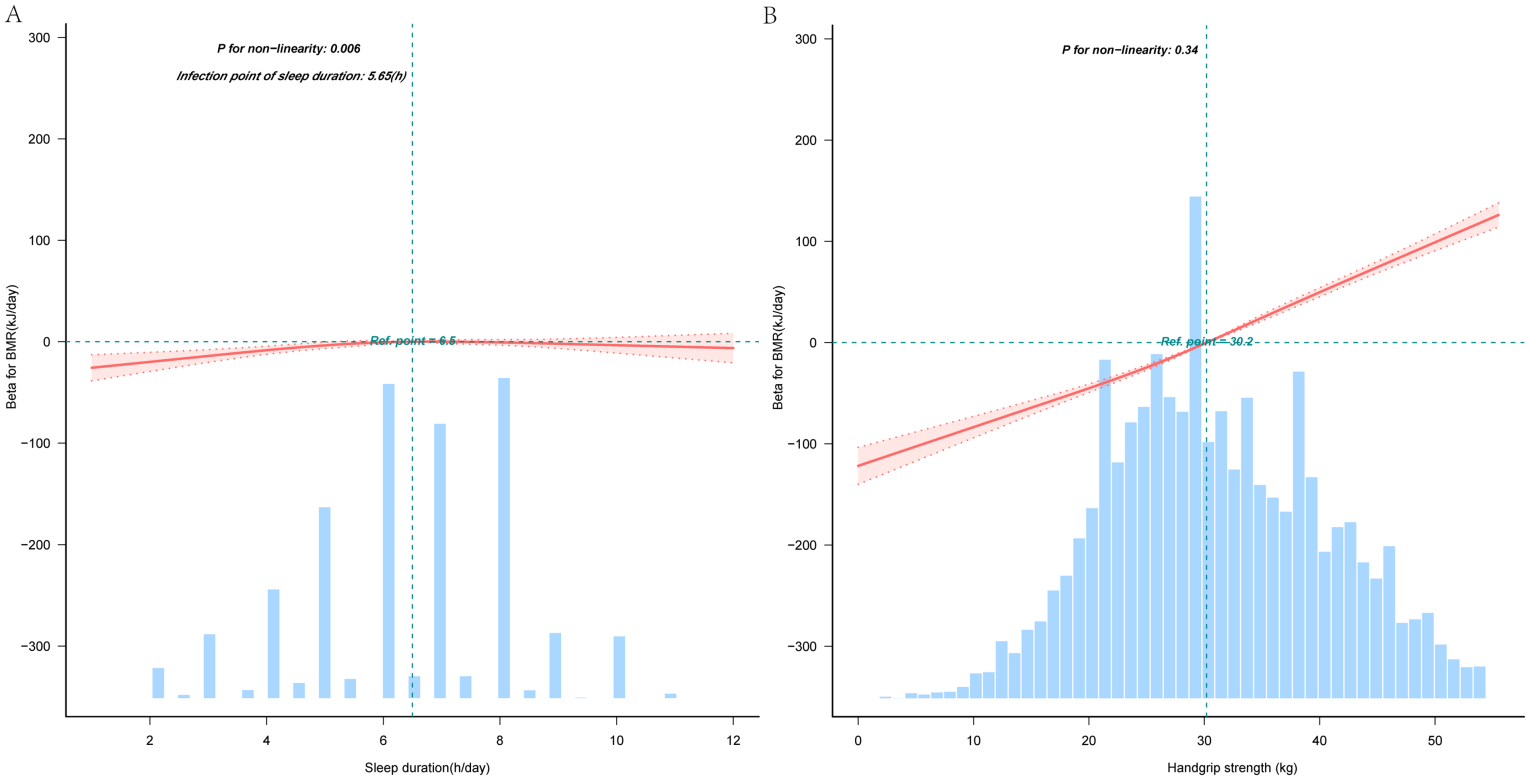
Nonlinear associations of sleep duration with basal metabolic rate **(A)**, nonlinear associations of handgrip strength with basal metabolic rate **(B)**. Solid and dashed lines represent the predicted value and 99% CI, respectively. The reference line (blue dot-dashed line) represents the median. Adjusted for age, gender, educational level, marital status, residence, smoking status, drinking status, BMI, and 14 chronic diseases, only 99% of the data is displayed. BMR, Basal metabolic rate.

The RCS regression analysis indicated a nonlinear relationship between sleep duration and BMR, with a significant nonlinearity (*p* = 0.006; see Figure 2A). But, Figure 2B illustrates that no non-linear relationship exists between grip strength and BMR. In the threshold analysis, the association of participants with sleep duration < 5.53 and BMR, *β* was 6.94 (95% CI: 3.48–10.40, *p* < 0.001) (Table 3). For sleep durations of 5.53 h or more, the link between sleep duration and BMR lacked statistical significance (95% CI: −2.96 - 0.95, *p* = 0.33).

**Table 3:** Threshold effect analysis of the relationship of sleep duration with basal metabolic rate.

Sleep duration	Adjusted model	
	*β* (95% CI)	*p* value
< 5.53	6.98 (3.51 ~ 10.45)	<0.001
≧5.53	−0.92 (−2.98 ~ 1.14)	0.38
Likelihood ratio test		<0.001

Adjusted for age, gender, educational level, marital status, residence, smoking status, drinking status, BMI, and 14 chronic diseases, only 99% of the data is displayed. OR, odds ratio; 95% CI, 95% confidence interval. *β*, Standardized regression coefficients; CI, confidence interval; BMR, basal metabolic rate.

### 3.3 Handgrip strength mediated the association between sleep duration and BMR

**Figure 3:**
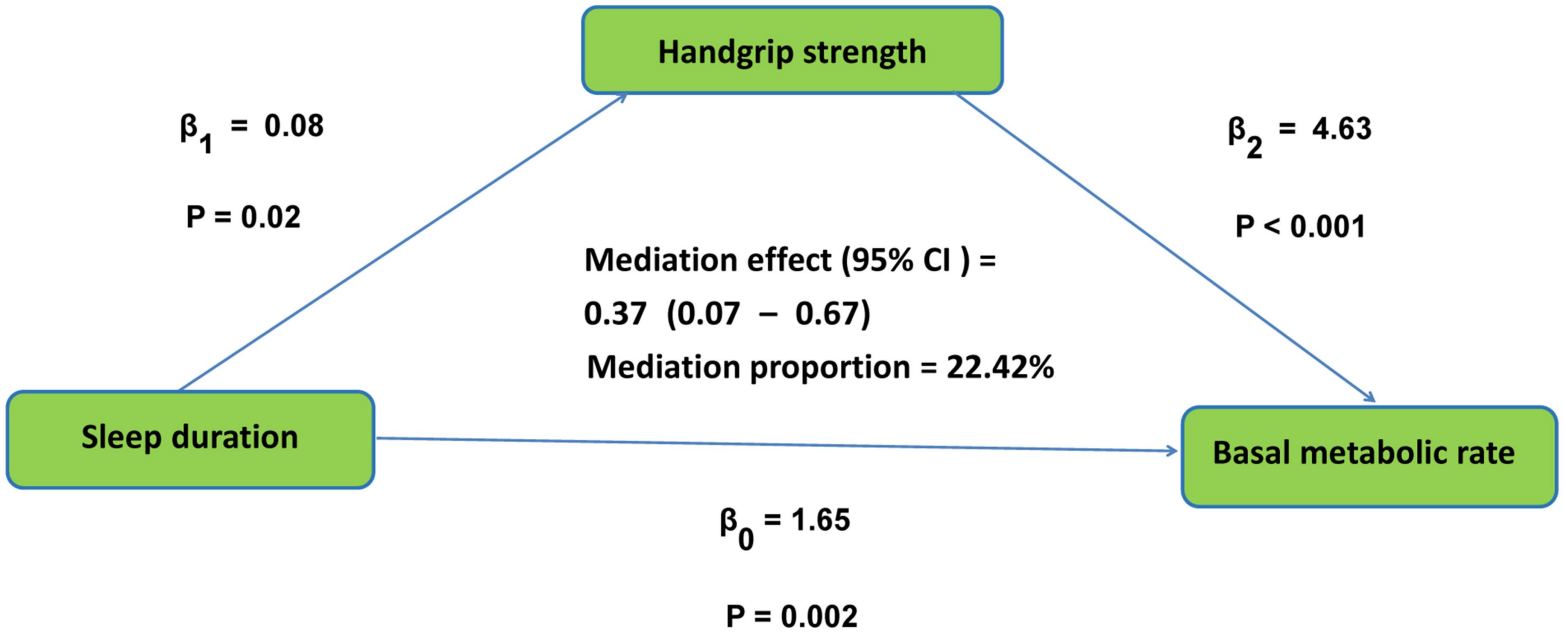
The conceptional framework of the mediation models. *β*_0_ was the total effect of sleep duration on basal metabolic rate; *β*_1_ represents the effect of sleep duration on handgrip strength; β_2_ represents the effect of handgrip strength on basal metabolic rate. The mediation effect was computed as the product of “*β*_1_” and “*β*_2_”(*β*_1_ × *β*_2_), and the mediation proportion was calculated as the ratio of the mediation effect product to total effects [(*β*_1_ × *β*_2_)/*β*_0_].

Table 4 shows the relationship between sleep duration, handgrip strength, and BMR. The study found a positive correlation between sleep duration and BMR (*r* = 0.11, *p* < 0.001). In addition, sleep duration was positively correlated with handgrip strength (*r* = 0.10, *p* < 0.001), and handgrip strength was positively correlated with BMR (*r* = 0.73, *p* < 0.001). By bootstrap analysis, the total effect of sleep duration on BMR was determined as 1.65, *p* = 0.002. Handgrip strength played a may mediate role between sleep duration and BMR, the size of the mediating effect was 0.37, 95% CI (0.07–0.67), accounting for 22.42% of the total effect of sleep duration on BMR, and the may mediate pathway is shown in Figure 3.

**Table 4:** Correlations among sleep duration, handgrip strength and basal metabolic rate.

Variables	Sleep duration	Handgrip strength	Basal metabolic rate
Sleep duration	1.00		
Handgrip strength	0.10***	1.00	
Basal metabolic rate	0.11***	0.73***	1.00

*** *p*-value < 0.001.

## 4 Discussion

This study is the first to explore how handgrip strength mediates the relationship between sleep duration and BMR in a cohort of middle-aged and older Chinese adults. The findings revealed a significant association between sleep duration and BMR. Furthermore, handgrip strength was found to partially may mediate this relationship, thereby supporting our initial hypothesis.

Research indicates a significant relationship between sleep duration and BMR, with studies highlighting the complex roles between sleep and metabolic processes. A study examined the impact of short sleep duration on energy metabolism, revealing that although it does not significantly alter total daily energy expenditure, it may influence specific components like resting metabolic rate and substrate utilization (17). This indicates that sleep duration could potentially affect BMR, given that RMR is a part of BMR. Furthermore, research on calorie restriction-like effects of resveratrol supplementation in obese humans demonstrated that resveratrol significantly reduced sleeping and resting metabolic rates, indicating that interventions affecting metabolic rate can be influenced by sleep-related factors (33). This study underscores the potential for sleep duration to modulate metabolic processes, including BMR. Finally, a study found that both long or short sleep duration were associated with increased risk of metabolic syndrome, which includes components such as altered glucose metabolism and obesity, both of which affect BMR (35). These studies show that sleep duration is closely connected to BMR and overall metabolic health, emphasizing the need for sufficient, quality sleep to maintain energy balance and metabolic function.

Sleep duration and handgrip strength have also been linked in multiple studies, underscoring the complex roles between sleep and physical health. One study examined the relationship between sleep duration and handgrip strength and found that both short and long sleep duration were associated with decreased handgrip strength, suggesting that optimal sleep duration is critical for maintaining muscle health (36). Similarly, another study found that sleep duration and quality were associated with muscle function, including handgrip strength, in older adults. Poor sleep quality and efficiency lead to decreased handgrip strength, highlighting the importance of good sleep hygiene for muscle maintenance (21). In addition, studies have shown that sleep duration affects metabolic and hormonal processes critical for muscle maintenance. Irregular sleep patterns can lead to hormonal imbalances that can affect muscle strength and recovery, including handgrip strength (37).

Handgrip strength, a measure of muscle strength, has proven significant association with BMR in older adults. For instance, a study conducted on older Koreans found a positive association between handgrip strength and BMR. The research indicated that higher handgrip strength was correlated with an increased BMR, suggesting that muscle strength exercises could be beneficial for regulating BMR in the older population (38). Meanwhile, another Korean study highlighted the relationship between handgrip strength and demographic factors, showing that handgrip strength was positively correlated with weight and height, which are key determinants of BMR (39). Additionally, the relationship between handgrip strength and physical performance has been explored in various populations. For instance, a study on older adults with type 2 diabetes found that handgrip strength relative to waist circumference was a strong predictor of mobility, which is closely linked to metabolic rate and energy expenditure (40). This underscores the potential of grip strength as an indicator of metabolic efficiency. Moreover, handgrip strength has been associated with cardiovascular health, which can influence metabolic rate. A study from the Korean Genome and Epidemiology Study found that handgrip strength was inversely related to arterial stiffness, a factor that can affect cardiovascular efficiency and, consequently, BMR (41). In summary, handgrip strength is a valuable measure that is associated with BMR through its links to muscle mass, physical performance, cardiovascular health, and overall metabolic efficiency.

Addressing poor sleep quality, low handgrip strength, and abnormal basal metabolic rate in middle-aged and older individuals necessitates a comprehensive strategy focusing on lifestyle modifications, physical activity, and nutritional interventions. Firstly, improving sleep quality is crucial as it is linked to various health outcomes. Exercise training programs moderately improve sleep quality in middle-aged and older adults. Programs involving moderate-intensity aerobic or high-intensity resistance exercises can decrease sleep latency and medication usage (42). Furthermore, music therapy, particularly listening to sedative music for a minimum of 4 weeks, enhances sleep quality in older adults (43). Regular physical activity also positively impacts handgrip strength, an indicator of overall health and mortality risk. Maintaining a healthy diet is crucial, as it correlates with increased muscle mass in older men (44). Additionally, interval walking training is a beneficial exercise that enhances physical fitness and may improve BMR in older adults (45, 46). Nutrition is vital in managing BMR. A balanced diet that includes adequate protein intake is essential for muscle maintenance and repair, which in turn supports a higher metabolic rate.

This study possesses several limitations. First, the self-reported sleep duration data may be subject to recall bias. Second, patients with missing data on the collected variables were excluded, which led to inevitable selective bias. Third, the cross-sectional study design imposes limitations on understanding the causality of the mediated moderation effect model. Thus, future research should employ a longitudinal study design to verify the causal direction of our mediated moderation model. Fourth, the study did not assess the severity and duration of grip strength loss, highlighting the need for further research. Fifth, our findings may have limited generalizability, because our study included only Chinese adults aged 45 years and above, this limitation may limit the relevance of our results to other ethnicities or global populations. Sixth, BMR is not measured directly but estimated using the Mifflin-St Jeor equation. Although the accuracy of this method is slightly higher compared with other methods, it is necessary to further study the directly measured BMR level.

## 5 Conclusion

The study found significant positive correlations between sleep duration and BMR, and between handgrip strength and BMR. Handgrip strength serves as a mediator between sleep duration and BMR. These findings are crucial for clinical practice and public health strategies aimed at preventing and managing BMR abnormalities in middle-aged and older adults.

## Data Availability

The original contributions presented in the study are included in the article/Supplementary material, further inquiries can be directed to the corresponding author.
